# The Impact of Undetected Hyperglycaemia During Pregnancy on Maternal and Neonatal Outcomes

**DOI:** 10.34763/jmotherandchild.20242801.d-24-00004

**Published:** 2024-10-23

**Authors:** Olga Olszak, Jarosław Kalinka

**Affiliations:** Department of Perinatology, I Division of Gynecology and Obstetrics, Medical University of Lodz, Poland; Department of Perinatology, Medical University of Lodz, Poland

**Keywords:** Diabetes, Gestational, Glucose Tolerance Test, Pregnancy, Hyperglycaemia

## Abstract

**Background:**

Despite clear diagnostic criteria for hyperglycaemia first detected in pregnancy, many pregnant women do not have the proper diagnosis. The following paper analyses the course of the perinatal period in pregnant women with undetected hyperglycaemia and their newborns.

**Materials and methods:**

Medical data of patients hospitalized in the Department of Perinatology between 2020 and 2022 was verified: 1st group: 40 patients with undetected hyperglycaemia, 2nd group: 40 with the diagnosis of gestational diabetes during pregnancy and adequate therapeutic management. The course of the perinatal period, abnormalities in the oral glucose tolerance test (OGTT) and the compliance with recommended postpartum tests were analysed.

**Results:**

There were significant differences in the newborn weights (p=0.039) – in the 1st group 15% large for gestational age (LGA) vs. 0% in the 2nd, and the occurrence of neonatal hyperbilirubinemia requiring phototherapy (p=0.007) – 22.5% in the 1^st^ group vs. 2.5% in the 2^nd^. The most common mistake in the OGTT was evaluation of fasting plasma glucose. In the 1^st^ group, no effect on incidence of hypertensive disorders, time or the route of delivery was observed. 75% from the 1^st^ group and 36% from the 2^nd^ did not perform postpartum OGTT (p=0.003).

**Conclusion:**

Hyperglycaemia in pregnancy is often undetected, which has a negative impact, especially on the neonates. In our study, LGA and hyperbilirubinaemia were significantly more common in neonates of mothers with undetected hyperglycaemia. These women had significantly more careless attitude to the postpartum diagnostic, which may influence future health and course of subsequent pregnancies. New and more effective methods of educating practitioners need to be implemented.

## Introduction

Despite clear diagnostic criteria and classification of hyperglycaemia first detected in pregnancy, many pregnant women are still admitted to the hospital during the perinatal period without being properly diagnosed with gestational diabetes mellitus (GDM). Although cared for by an obstetrician and undergoing appropriate diagnostic tests, these patients have not been properly diagnosed with GDM, and no further therapeutic procedures have been initiated. In our study, we focused on pregnant patients who, during hospitalisation in the Department of Perinatology for various reasons, had GDM screening results from pregnancy reviewed that showed previously unidentified abnormalities and undetected hyperglycaemia in pregnancy. The aim was to analyse how incorrect test results would affect the patient’s perinatal period and the newborn’s condition.

Diagnosing hyperglycaemia in pregnancy starts with the first prenatal visit, during which fasting plasma glucose (FPG) is ordered. If the result is correct, i.e. <92 mg/dl (<5.1 mmol/l), an oral glucose tolerance test (OGTT) should be scheduled between 24 and 28 weeks of pregnancy. If the FPG result is 92–125 mg/dl (5.1 mmol/l – 6.9 mmol/l), the test should be performed immediately, and if OGTT is normal, it should be repeated at 24–28 weeks of gestation. When at least one value of OGTT exceeds the standards according to World Health Organization (WHO) 2013 recommendations, we recognise hyperglycaemia in pregnancy. The diagnostic criteria for GDM in OGTT are FPG 92–125 mg/dl (5.1–6.9 mmol/l), 1-hour plasma glucose ≥ 180 mg/dl (10.0 mmol/l) and 2-hour plasma glucose 153–199 mg/dl (8.5–11.0 mmol/l). Diabetes in pregnancy (DIP) should be identified if one or more of the following criteria are met: FPG ≥ 126 mg/dl (7.0 mmol/l), 2-hour plasma glucose ≥ 200 mg/dl (11.1 mmol/l) during OGTT, random plasma glucose ≥ 200 mg/dl (11.1 mmol/l) in the presence of diabetes symptoms (WHO). For women who have specific risk factors such as a pre-pregnancy BMI of 30 kg/m^2^ or higher, a history of GDM, a positive family history of type 2 diabetes in female first-degree relatives, or have delivered a newborn weighing 4500g or more, it is recommended to order an OGTT at the beginning of pregnancy [1].

Since the Hyperglycaemia and Adverse Pregnancy Outcome (HAPO) study and subsequent WHO recommendations were published, the diagnostic criteria and classification of hyperglycaemia first detected in pregnancy have become more evident. Practitioners received precise guidelines regarding timing and conditions for conducting OGTT and interpretation of the results. The cut-off values for FGP, 1-hour and 2-hour plasma glucose are commonly known by practitioners. Moreover, the criteria were approved by the International Association of the Diabetes and Pregnancy Study Groups (IADPSG) in 2010, by WHO in 2013, and by the Polish Diabetes Association in 2015, and have not been modified subsequently.

The following paper analyses cases of patients with undetected hyperglycaemia hospitalized in the Department of Perinatology in the 3rd level perinatal centre between 2020 and 2022. The diagnosis was made after a thorough analysis of the patient’s documentation from the time of pregnancy and identification of abnormalities in GDM screening tests. We investigated the course of the perinatal period of pregnant women and their newborns. We compared these patients with a control group of pregnant women diagnosed properly with GDM during pregnancy and with adequate therapeutic management. Moreover, we conducted an analysis of mistakes performed during OGTT interpretation. Additionally, compliance with recommended postpartum tests was analysed.

## Materials and methods

We analysed medical documentation of patients hospitalised in the Department of Perinatology between 2020 and 2022. 40 patients with undetected hyperglycaemia were found (referred to as 1st group). This was possible due to the analysis of laboratory test results performed during pregnancy and obtained from patients’ medical records. Abnormalities in the OGTT were identified. Next, perinatal data, including operative deliveries and caesarean sections, were investigated. Information about newborns – birth weight, hypoglycaemia and hyperbilirubinaemia in the postpartum period – was obtained. We also contacted the patients and inquired if they followed the postpartum hyperglycaemia diagnostic instructions provided upon discharge from the hospital.

We collected the same data from 40 pregnant women who had been properly diagnosed with GDM according to Polish Society of Gynaecologists and Obstetricians recommendations, had adequate therapeutic management implemented during pregnancy and were hospitalized in our department in the same period (2^nd^ group). We compared the group of women with undetected hyperglycaemia (1^st^ group) with the group diagnosed with GDM (2^nd^ group) regarding the perinatal course in the mother and newborn, as well as compliance with postnatal diagnostics.

Obtained continuous data was presented as mean and standard deviation, whereas categorical data was presented as numbers and percentages. We used the chi-square test for comparison of categorical data between two groups and students unpaired ‘t’ for comparison of continuous data between two groups. A *P* value of less than 0.05 was considered significant.

## Results

**Figure 1.**
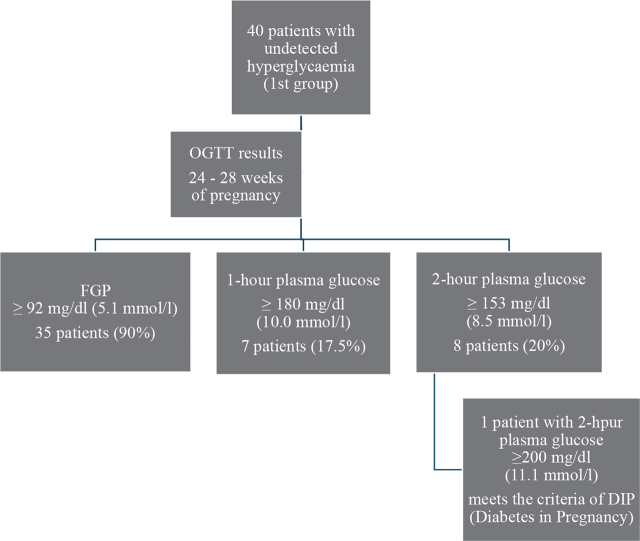
The results of the 75 g oral glucose tolerance test in the group of pregnant patients with undetected hyperglycaemia (1^st^ group).

We have found 40 patients with undetected hyperglycaemia (1st group) hospitalised between 2020 and 2022. In this period, the rate of undetected hyperglycaemia in the group of all patients with GDM was 5.5% (40/722). The results of OGTT (Fig. 1) performed in the reference period (24–28 weeks of pregnancy) were analysed in the 1^st^ group (undetected hyperglycaemia). 35 out of 40 (90%) patients had an abnormal FPG prior to 75g oral glucose load, including 6 patients with plasma glucose levels ≥ 100 mg/dl (5.5 mmol/l). 7 patients had high 1-hour plasma glucose, while 8 had elevated 2-hour plasma glucose levels. 8 patients had 2 or more OGTT abnormalities. In one patient, the OGTT results (fasting, 1-hour and 2-hour, 105, 225 and 203 mg/dl, respectively) indicated diabetes in pregnancy (DIP). We do not have the OGTT results in the 2nd group of patients (with GDM monitored and treated during pregnancy).

The data on maternal and neonatal outcomes were compared with randomly selected 40 patients diagnosed and treated for GDM during pregnancy (2^nd^ group) and hospitalised between 2020 and 2022 in our department. 29 patients (72.5%) had diabetes that was controlled with a diet (GDM - G1). 11 patients (27.5%) required insulin to achieve normal plasma glucose values (GDM - G2).

The mean age of the patients in the 1st and 2nd groups was 32.8 years and 32.0 years, respectively (p=0.536). There was no significant difference in patients’ Body Mass Index (BMI) between the two groups (p=0.157). More women in the 2nd group were primigravida (57.5% vs. 35%). Table 1 compares patients in both groups.

**Table 1.** The comparison of course of pregnancy, neonatal and maternal outcomes in both groups – 1^st^ group with undetected hyperglycaemia, 2^nd^ group with GDM during pregnancy and adequate therapeutic management.

		**1^st^ group (n=40)**	**2^nd^ group (n=40)**	***P* value**
Characteristics of pregnant patients:				
Age in years – mean (SD)		32.8 (5.14)	32.0 (5.31)	0.536
BMI (kg/m^2) – mean (SD)		31.8 (5.30)	31.4 (5.50)	0.743
Primigravida – n (%)		14 (35)	23 (57.5)	0.044
Multigravida – n (%)		26 (65)	17 (42.5)	
Maternal outcomes:				
Hypertensive disorders during pregnancy - hypertension, preeclampsia, HELLP (%)		7 (17.5)	7 (17.5)	1.00
Time of delivery – weeks of gestation		39+2	39+1	0.787
The route of delivery:	Elective caesarean section	5	12	0.121
	Emergency caesarean section	12	14	
	Vaginal delivery	22	14	
	Instrumental delivery (forceps)	1	0	
Neonatal outcomes:				
Neonatal weight	SGA – n (%)	4 (10)	5 (12.5)	0.039
	AGA – n (%)	30 (75)	35 (87.5)	
	LGA – n (%)	6 (15)	0 (0)	
Hyperbilirubinaemia – n (%)		9 (22.5)	1 (2.5)	0.007
Hypoglycaemia <40 mg/dl – n (%)		3 (7.5)	5 (12.5)	0.456

Subsequently, we analysed maternal outcomes in both groups. There was no significant difference in the occurrence of hypertensive disorders during pregnancy, time of delivery nor the delivery route in patients from the 1^st^ and 2^nd^ group (Table 1).

However, we have found some differences regarding neonates of patients with undetected hyperglycaemia and with GDM during pregnancy and adequate therapeutic management (Table 1). There were significant differences in the newborns’ weight from the 1^st^ and 2^nd^ groups (p=0.039). In the 1^st^ group, there was 10% small for gestational age (SGA - body weight <10 pc for gestational age) and 15% large for gestational age (LGA - body weight >90 pc for gestational age) newborns, whereas in the 2^nd^ group 12.5% and 0%, respectively. Newborns with appropriate weight for gestational age (AGA) constitute 75% and 87.5% of the 1^st^ and 2^nd^ group, respectively. Moreover, there were differences in the occurrence of significant neonatal hyperbilirubinemia requiring phototherapy found in the first days after birth (p=0.007) – 9 newborns of mothers from the 1st group and 1 newborn of a mother from the 2^nd^ group. 3 newborns of mothers from the 1^st^ group experienced hypoglycaemia lower than 40 mg/dl, while none had hypoglycaemia lower than 35 mg/dl. In the 2^nd^ group, hypoglycaemia was found in 5 newborns (5 when <40 mg/dl, including 3 <35 mg/dl), so the differences were not significant (p=0.456).

Postpartum, we contacted and obtained information from 28 patients (70%) from both groups. As many as 21 out of 28 (75%) patients from the 1^st^ group (undetected hyperglycaemia) did not visit a diabetologist or perform OGTT 6 weeks after delivery - the above instructions were given to the patients on their discharge card from the hospital. 7 patients performed a glucose tolerance test and received correct results. Out of the 2^nd^ group (with GDM during pregnancy and adequate therapeutic management), 18 women (64%) underwent testing, and 16 received correct results. One was diagnosed with type 2 diabetes, and one was prediabetic with impaired fasting glucose. 10 of 28 patients from the 2^nd^ group did not perform postpartum diagnostic tests, but many emphasised that they remembered the obligation to perform the test and would undoubtedly do it soon. There was a significant difference in the approach to performing tests in the postpartum period between patients from group 1st and group 2^nd^ (p=0.003) – Table 2.

**Table 2.** The compliance with recommended diagnostics in postpartum period in patients from both groups.

	**1^st^ group (n=28)**	**2^nd^ group (n=28)**	***P* value**
Did not perform the OGTT (%)	21 (75)	10 (36)	0.003
Performed the OGTT (%)	7 (25)	18 (64)	

## Discussion

We decided to explore the problem of undetected hyperglycaemia in pregnancy because the number of patients admitted to our hospital without proper diagnosis is high and still growing - in 2020, we had 7 such patients, in 2021, 17 and 16 in 2022. It is essential to consider how the absence of proper diabetic care and treatment can affect the mother’s and newborn’s health. If appropriate management had been implemented sooner, certain perinatal complications and failures may have been prevented.

Despite the small size of the two groups, the results indicate some important differences. Patients with undetected hyperglycaemia (1^st^ group) more often gave birth to LGA newborns (6 vs 0) – p = 0.039. Moreover, neonates of these mothers more often suffered from hyperbilirubinaemia (9 vs 1) – p=0.007. Furthermore, patients with undetected hyperglycaemia (1^st^ group) less frequently performed postpartum diabetes diagnostic tests (p=0.009).

Implementation of treatment (dietary, pharmacological) in the case of GDM has a large impact on the course of the perinatal period. Undetected and untreated GDM is associated with severe perinatal complications. A study conducted by Hunger-Dathe et al. in 2005 shows that mothers with untreated GDM more often give birth to LGA newborns. This group also has an elevated prevalence of preeclampsia. Treatment of GDM can reduce the likelihood of hyperinsulinaemia, postnatal hypoglycaemia, and jaundice requiring treatment in neonates. Additionally, treatment of GDM can help prevent diabetic fetopathy in macrosomic infants [2]. These results are adequate to the results of our study - the number of LGA neonates was significantly higher in the 1st (untreated hyperglycaemia) than in the 2^nd^ group. Moreover, the number of newborns with jaundice requiring treatment was significantly higher in the 1st group. However, in our study we did not find hypoglycaemia less common in neonates of treated mothers – neonatal hypoglycaemia was insignificantly more frequent in the 2nd group. In our analysis the percentage of hypertension in pregnancy was equal in both groups, but there were 4 cases of preeclampsia in the 1st group. Of course, limited number of participants does not allow for unambiguous conclusions, but it may indicate a general trend confirmed by other scientific studies.

Regarding the question of LGA neonates of mothers with GDM, worth noticing are the results of a meta-analysis conducted by Poolsup et al. in 2014, which indicate that the initiation of therapy in GDM reduces the risk of LGA [3]. Still, it may not necessarily have a long-term positive impact on the child’s weight. Gillman, in his study from 2010, found that although GDM treatment significantly reduced the risk of LGA, it did not affect the child’s BMI from 4 up to 5 years [4].

Crowther et al. showed that treatment of GDM reduces serious perinatal morbidity and may also improve women’s health-related quality of life. Serious perinatal complications were significantly more common among the infants of mothers without proper diabetic intervention (dietary advice, blood glucose monitoring, and insulin therapy if needed). Pregnant women with GDM and described above diabetic intervention had a higher rate of labour induction, but there was no difference in the rate of caesarean sections. Data collected three months postpartum revealed lower rates of depression and improved health status in the group with diabetic intervention [5].

Behboudi-Gandevani et al. published a systemic review and meta-analysis in 2021 about the influence of diet-controlled GDM on adverse pregnancy outcomes. The treatment of diet-controlled GDM significantly reduced the risk of LGA, shoulder dystocia, caesarean section, preeclampsia, elevated cord C-peptide, and respiratory distress syndrome compared to untreated GDM patients [6].

Transient hypoglycaemia is common in neonates shortly after birth. In maternal hyperglycaemia, glucose freely crosses the placenta with a concentration gradient. Insulin does not cross the placenta but is produced by the foetal pancreas from the early stage of pregnancy. Foetal hyperinsulinaemia predisposes to neonatal hypoglycaemia. In hypertrophic infants of diabetic mothers, hypoglycaemia may result from insufficient glycogen storage [7]. Theoretically, neonatal hypoglycaemia should be less severe in pregnant women with GDM and proper treatment. In our study, neonatal hypoglycaemia was insignificantly more frequent in the 2nd group. It may result from insufficient glycaemia control in GDM patients earlier in pregnancy.

Around 15% of neonates born to mothers with GDM develop hyperbilirubinaemia [8]. Our study found that significantly more neonates from 1^st^ group experienced hyperbilirubinaemia requiring phototherapy in the first few days after birth. He et al., in their study from 2022, conclude that hyperbilirubinaemia may indicate abnormal placental morphology and poor control of maternal glycaemia in patients with GDM [9].

The reason of incorrect interpretation of the OGTT results was investigated. Possible explanations include:

– The use of diagnostic cut-offs following WHO recommendations from 1999, but with these criteria GDM would be diagnosed in 8 patients.
– The use of Polish Society of Gynaecologists and Obstetricians recommendations from 2011 (partially considered the results of the HAPO study) - GDM would be diagnosed in 15 pregnant women.
– The use of diagnostic criteria for diabetes in the general population – DM (diabetes mellitus) would be diagnosed in 1, IFG in 6, and IGT (impaired glucose tolerance) in 8 pregnant women.
– The lack of knowledge of the current diagnostic criteria for GDM, included in WHO recommendations from 2013 and Polish Society of Gynaecologists and Obstetricians recommendations in 2017 (they have not been modified since then).

Table 3 presents diagnostic criteria for GDM in selected recommendations.

**Table 3.** Diagnostic criteria for GDM in OGTT included in selected recommendations.

WHO 1999	GDM	Criteria for DM	Fasting ≥ 126 mg/dl (7.0 mmol/l)
			or
			2-h post glucose ≥ 200 mg/dl (11.1 mmol/l)
		Criteria for IGT	Fasting < 126 mg/dl (7.0 mmol/l)
			and
			2-h post glucose ≥ 140 mg/dl (7.8 mmol/l)
PGS 2011	GDM	One of the following:	Fasting ≥ 100 mg/dl (5.6 mmol/l)
			1-h ≥180 mg/dl (10.0 mmol/l)
			2-h ≥ 140 mg/dl (7.8 mmol/l)
WHO 2013 PGS 2015	GDM	One of the following:	Fasting 92 – 125 mg/dl (5.1 mmol/l)
			1-h ≥ 180 mg/dl (10.0 mmol/l)
			2-h ≥ 153 mg/dl (8.5 mmol/l)

Incorrect diagnostic management of gestational diabetes has already been analysed by Polish authors. The aim of the study conducted by Molęda et al. in 2015 was to assess the practical implementation of Polish Society of Gynaecologists and Obstetricians standards of GDM screening and diagnosis. They analysed 351 patients between 2008 and 2010 (recommendations from 2005 were in force) and later 249 patients between 2011 and 2013 (recommendations 2011). Adherence to the diagnostic guidelines in the study’s first phase was 42.2%, and in the later stage - 78.3% [10].

Imoh et al. emphasise the problem of incorrect screening in their 2022 study. Antenatal healthcare providers had to complete a semi-structured, self-administered questionnaire, and data were analysed for adherence to recommended screening practices such as WHO, IADPSG and NICE (National Institute for Health and Care Excellence) guidelines. Most of the 128 respondents (68%) screened all pregnant women (universal screening) for GDM. FPG (77%) and random blood glucose (56%) were the most common tests. Only 27 respondents (22 %) were using OGTT to detect GDM [11].

In the study from 2015, Babu et al. showed that the knowledge of GDM among Indian doctors was insufficient. 50 gynaecologists took part in a survey based on a semi-structured questionnaire. 12% of them provided the correct answers regarding the general understanding of GDM - diagnosis and management. 20% knew postpartum recommendations of GDM. The majority (92%) had poor knowledge about the cut-off values of glucose tests. 46% based their diagnosis on a random blood glucose test [12].

From the 1^st^ group, only a quarter of patients (25%) repeated the OGTT postpartum. Significantly more women (64%), but still not all, performed the recommended examination in the 2^nd^ group. Unfortunately, this is a common occurrence in women with GDM. In a study conducted by Gennaro et al. analysing the behaviour of 650 women, only 41% performed the glucose tolerance test as recommended. DM among them was diagnosed in 2% of women. The screening was performed less frequently by women with a family history without type 2 diabetes, young women, i.e. <35 years old, with a lower education level and unstable employment [13].

Women with gestational diabetes have a higher risk of developing impaired glucose regulation and diabetes in the future, and being overweight seems to be the most important risk factor. Therefore, this group of patients should be given additional care. Risk factors for diabetes and prediabetes 6–12 weeks after delivery include: GDM before 24 weeks of gestation, degree of hyperglycaemia at diagnosis, insulin requirement, HbA1c in the month preceding delivery, age >36 years, family history of diabetes, preterm delivery [14].

We should consider what actions can be implemented to improve diagnostics of hyperglycaemia in pregnancy and reduce the number of patients with undetected GDM. An attempt to solve the problem may be creating an appropriate program or application in which the doctor would enter the patient’s data and the results of individual tests. The algorithm would indicate the next steps for gynaecologists and make it easier to confirm a diagnosis according to the current guidelines. Korvesi et al. described such an attempt in 2020 [15].

Our study drew attention to the problem of undetected hyperglycaemia in pregnancy, which has an impact on the well-being of the foetus and the mother. Nevertheless, a significant group of pregnant women is not properly diagnosed. Despite the small size of both groups, we were able to present some important conclusions.

The study indicates differences in foetal growth disorders. LGA was significantly more common in neonates of mothers with undetected hyperglycaemia. Moreover, they more often underwent phototherapy due to hyperbilirubinemia. There was no significant effect on the occurrence of hypoglycaemia in the newborn.

In pregnant women with undetected hyperglycaemia, no effect on the incidence of hypertensive disorders during pregnancy, time of delivery or on the route of delivery was observed. However, the results indicate a significantly more careless attitude to the postpartum diabetic tests. The patients with undetected hyperglycaemia were significantly less likely to perform the recommended postpartum oral glucose tolerance test. Failure to diagnose diabetes or prediabetes may significantly impact a woman’s future health and the course of her subsequent pregnancies.

The lack of proper diagnosis of hyperglycaemia during pregnancy may be related to the lack of knowledge of current diagnostic criteria by practitioners or their dismissive approach. It is essential to prioritise new education methods and look for new ways to keep obstetricians updated on the latest knowledge and recommendations.

### Key points

- Diagnostics of gestational diabetes (GDM) is still not properly performed and World Health Organization (WHO) diagnostic criteria are not sufficiently applied.
- The lack of proper diagnosis of hyperglycaemia in pregnancy has a negative impact on the course of the perinatal period in the mother and her newborn.
- Patients with undetected hyperglycaemia are significantly less likely to perform the recommended postpartum oral glucose tolerance test (OGTT), which may adversely affect a woman’s future health and the course of her subsequent pregnancies.
